# DeviceTalk: A No-Code Low-Code IoT Device Code Generation

**DOI:** 10.3390/s22134942

**Published:** 2022-06-30

**Authors:** Whai-En Chen, Yi-Bing Lin, Tai-Hsiang Yen, Syuan-Ru Peng, Yun-Wei Lin

**Affiliations:** 1 Department of Computer Science and Information Engineering, Asia University, Taichung City 413305, Taiwan; wechen@asia.edu.tw; 2 China Medical School, College of Humanities and Sciences, Taichung City 40402, Taiwan; 3 Miin Wu School of Computing, National Cheng Kung University, Tainan City 70101, Taiwan; 4 College of Computer Science, National Yang Ming Chiao Tung University, Hsinchu City 300093, Taiwan; ksoy.cs08g@nctu.edu.tw (T.-H.Y.); dz92286@gmail.com (S.-R.P.); 5 College of Artificial Intelligence, National Yang Ming Chiao Tung University, Hsinchu City 300093, Taiwan; jyneda@nctu.edu.tw

**Keywords:** no-code, low-code, code generator, IoT, actuator

## Abstract

The deployment of a client–server-based distributed intelligent system involves application development in both the network domain and the device domain. In the network domain, an application server (typically in the cloud) is deployed to execute the network applications. In the device domain, several Internet of Things (IoT) devices may be configured as, for example, wireless sensor networks (WSNs), and interact with each other through the application server. Developing the network and the device applications are tedious tasks that are the major costs for building a distributed intelligent system. To resolve this issue, a low-code or no-code (LCNC) approach has been purposed to automate code generation. As traditional LCNC solutions are highly generic, they tend to generate excess code and instructions, which will lack efficiency in terms of storage and processing. Fortunately, optimization of automated code generation can be achieved for IoT by taking advantage of the IoT characteristics. An IoT-based distributed intelligent system consists of the device domain (IoT devices) and the network domain (IoT server). The software of an IoT device in the device domain consists of the Device Application (DA) and the Sensor Application (SA). Most IoT LCNC approaches provide code generation in the network domain. Very few approaches automatically generate the DA code. To our knowledge, no approach supports the SA code generation. In this paper, we propose DeviceTalk, an LCNC environment for the DA and the SA code development. DeviceTalk automatically generates the code for IoT devices to speed up the software development in the device domain for a distributed intelligent system. We propose the DeviceTalk architecture, design and implementation of the code generation mechanism for the IoT devices. Then, we show how a developer can use the DeviceTalk Graphical User Interface (GUI) to exercise LCNC development of the device software.

## 1. Introduction

Internet of Things (IoT) development platforms for distributed intelligent systems have exponentially grown in industry-specific applications involving sensor tracking and monitoring. In such systems, IoT devices are an essential component. The IoT development platform examples of such distributed intelligent systems are oneM2M [1], IoTtalk [2] and so on. In these platforms, wireless sensor networks (WSNs) [3,4,5] are accommodated for communications, and the programs of a distributed intelligent application are developed in both the network domain and the device domain. In the network domain, a network application is created to be executed in the server (typically located in the cloud) following the oneM2M or IoTtalk network Application Programming Interface (API). In the device domain, the WSN nodes implement the device applications, which are equipped with specific oneM2M or IoTtalk drivers (software modules) to connect to the distributed intelligent system.

Developing the network applications and the device applications are tedious tasks that are the major costs for building a distributed intelligent system. Chris Wanstrath, the former CEO of GitHub, said that the mainstream trend for the design of future programs is “no-code”. The purpose of using low-code or no-code (LCNC) programming is to automate code generation during application development and to reduce the effort of developing and putting applications into production. According to the Gartner survey, more than 65% of enterprises will adopt no-code technology for digital transformation in 2024; at the same time, Global Newswire also believe that by 2030, the compound growth rate of the no-code platform will reach 31%, and the revenue will reach as much as 180 billion USD.

LCNC is particularly useful for the development of IoT-based smart applications that require information technology (IT) knowledge for integrating the IoT devices, the IoT servers, the communication gateways, databases, etc. Regular development for IoT setup may be difficult, but can be accelerated by LCNC tools. As traditional LCNC solutions are highly generic, they may not be able to directly address the intricacies of IoT infrastructure. In particular, automated code generation tends to generate excess code and instructions, which will lack efficiency in terms of storage and processing. Fortunately, optimization of automated code generation can be achieved for IoT by taking advantage of the IoT characteristics. Take IoTtalk, for example [2]; in this IoT application development platform, LCNC tools are provisioned for rapidly developing summary reports with drill down capability. Specifically, it automatically generates maps [6] and dashboards that can become the components of an integrated operations center (IOC) for IoT applications.

Furthermore, since LCNC programs are automatically generated, we can take advantage of the code generation rules to provide the mechanisms that guarantee these programs are made safe from failure. For example, IoTtalk offers the VerificationTalk mechanism [7] to assist in bulletproofing developers from inadvertently creating errors or vulnerabilities in their IoT applications.

Therefore, an LCNC IoT development environment is essential for people to create innovative IoT applications without much IT knowledge or any coding ability. Such an IoT platform automatically connects remote devices and enables the developers to track and manage smart applications with the utmost ease. In [8], the languages and tools supporting the development of IoT systems were surveyed to understand the state of the art of existing low-code platforms. By analyzing sixteen platforms, a corresponding set of features has been identified to represent the functionalities and the services that each analyzed platform can support. These features are described below.

**Figure 1:**
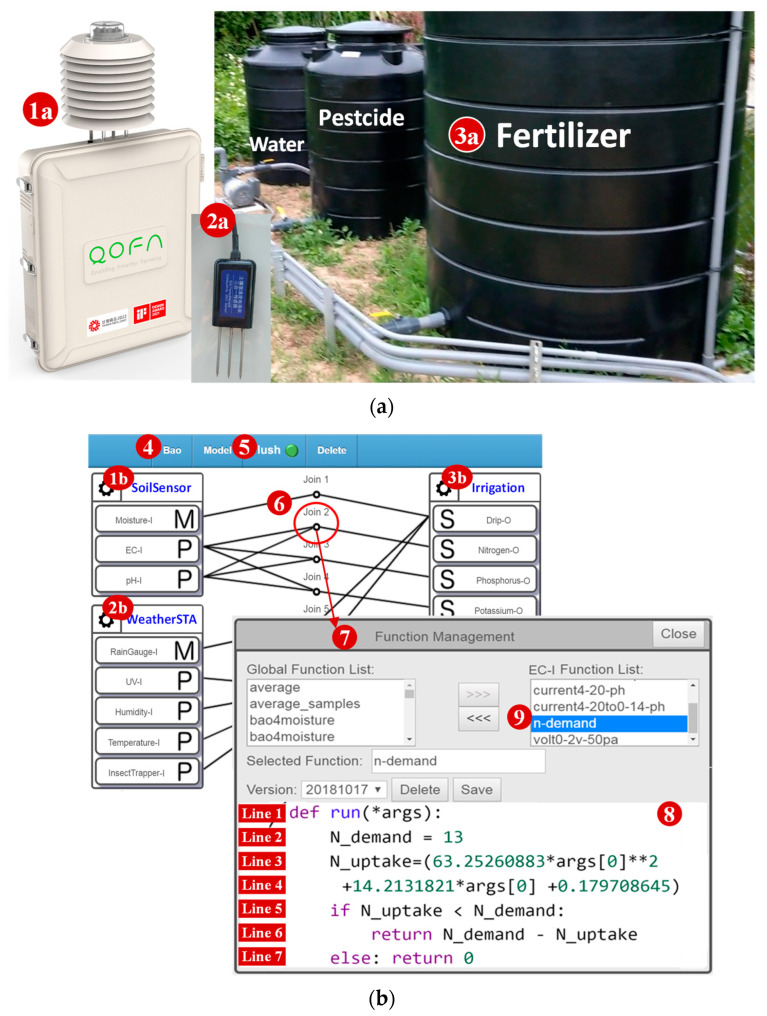
No-code approach for creating a smart agriculture application. (**a**) The physical hardware layout. (**b**) Application creation through LCNC.

Through a graphical user interface (GUI), a no-code approach enables non-programmers to build IoT applications by dragging and dropping graphical icons. These solutions provide a simple application development environment at the cost of less flexible features. An example is a smart agriculture application created by the IoTtalk GUI [2], illustrated in Figure 1. In this application, a micro weather station (WeatherSTA; Figure 1(1a)) and a soil sensor set (SoilSensor; Figure 1(2a)) are used to control the irrigation system (Figure 1(3a)), which is created by dragging lines between the WeatherSTA/SoilSensor icons (Figure 1(1b,2b)) and the Irrigation icon (Figure 1(3b)).

Figure 1(4) illustrates the Bao Farm application project developed through the “Project” GUI window, where the IoTtalk supports the developer to assemble pre-configured software modules to build the applications easily. Such modules can be accessed through the “Model” drop-down list (Figure 1(5)). The selected device models are shown in the project window as the icons (Figure 1(1b,2b,3b)).

With the drag-and-drop mechanism, the developer can conveniently create the functions needed and connect them (e.g., the join links in Figure 1(6)) into a logical chain to build the IoT applications. The visual modeling mechanism allows the developer to graphically convert innovation into workflows by dragging, dropping and assembling the icons without scripting code. The LCNC platform provides a basic user interface for connecting to a preferred database and seamlessly converts data models into relational tables. Also, as a GUI-based low-code integration approach, IoTtalk minimizes the coding complexity. The developers are not required to have the IoT technical knowledge, and only need to write a small number of codes to create their IoT applications. In the Bao Farm project, the developer wants to intelligently control the irrigation system based on the relationship between the electric conductivity (EC) value $\sigma_{b}$ and the Nitrogen value *f_N_*(*σ_b_*) for the Bao farm [9], which is expressed as

(1) $$f_{N}\left(\sigma_{b}\right)=63.2526\sigma_{b}{}^{2}+14.2131\sigma_{b}+0.1797$$

To control the Nitrogen dripper based on the EC sensor, we connect their icons by the link Join 2 (Figure 1(6)). Then, we click the circle in the middle of the link to pop up the “Function Manager” window (Figure 1(7)) and implement Equation (1) through a Python function (Figure 1(8)) where *args[0]* is the EC values received from the soil sensor (Figure 1(1b)). This function can be saved in IoTtalk as a software module (Figure 1(9)) to be used by other applications.

Most LCNC IoT approaches have focused on code generation in the network domain, which connects the IoT devices and manipulates the data/messages delivered among these devices [10,11,12,13,14,15]. These approaches assume that the software installed in an IoT device (the sensor logic and the driver for communications to the IoT server) already exists. Very few LCNC approaches have focused on software development for IoT devices [16]. In this paper, we propose DeviceTalk, an LCNC environment for IoT device software development. The paper is organized as follows. Section 2 surveys the related work. Section 3 proposes the DeviceTalk architecture. Section 4 designs and implements the code generation mechanism for the IoT devices. We show how a developer can use the DeviceTalk GUI to exercise LCNC development of the device software.

## 2. Related Studies

It is a tedious task to develop an IoT application and configure the sensor and the actuator devices. It becomes more imperative for the developers to create their IoT applications with minimal programming skills. As we mentioned, the software of an IoT application is developed in two domains. In the network domain, a network application is required to connect the IoT devices and manipulate the data transmitted among them. The network application is typically executed by an IoT server in the cloud. In the device domain, two software modules, i.e., sensor and actuator application (SA) and device application (DA), should be installed in an IoT device. The SA implements the logic for sensors, controls and/or actuators. The DA implements the driver to connect to the IoT server in the network domain. LCNC solutions in the network domain have been developed in the past, including the IoTtalk mechanisms described in Figure 1. Most IoT LCNC approaches provide code generation in the network domain. Very few approaches automatically generate the DA code. To our knowledge, no approach supports SA code generation. In this paper, we propose DeviceTalk, an LCNC environment for the DA and the SA code development.

In [17], the authors proposed a language called BIoTA (Buildout IoT Application Language) to assist and streamline the building of software architectures for IoT. BIoTA designs and implements a grammar and a compiler for syntax and semantic analysis, as well as code generation for IoT network applications. An integrated development environment (IDE) was implemented using the BIoTA language for reading and creating software architectures. With the BIoTA IDE, the authors demonstrated three examples of software architectures for public buildings, irrigation and parking.

In [18], the authors developed IoT network applications based on the formalism transformation graph (FTG) process model (PM) approach and described the model-driven engineering (MDE) process of developing applications for different platforms or operating systems. FTG + PM tackles the complexity of multi-paradigm systems using MDE to improve the usability, precision and automation of these systems. A platform-independent IoT model example of the irrigation system was given to demonstrate how FTG + PM works. The solution did not provide a friendly GUI like the one in Figure 1.

The study in [19] integrated the data analytics capabilities of Spark in IoT mashup tools with a wide range of data interfaces and application programming interfaces (APIs). The authors proposed aFlux, a graphical flow-based programming paradigm to analyze the Spark ecosystem with appropriate data interfaces. aFlux is a generic Spark programming approach based on graphical flows, which supports early-stage validation and code generation of Java Spark programs. aFlux was implemented as a Java Virtual Machine (JVM)-based mashup tool and was evaluated in three use cases to demonstrate the machine learning and stream analytics capabilities of Spark.

By using attribute-driven design and MDE, the study in [20] proposed an IoT application development framework called IADev. This framework first develops an iterative architecture using attribute-driven design. Specifically, it transforms the requirements into a solution architecture by considering the concerns of all stakeholders involved. Then, it uses MDE for generating models to guide the transformation. Specifically, the generated MDE metamodels hierarchically transform the design components into software artifacts. IADev was used to generate an executable implementation code for a smart vehicle scenario in an intelligent transportation system, and was used with the Siemens IoT cloud platform to perform service orchestration in industrial IoT.

In [21], the authors developed an MDE approach to generate code and develop IoT systems simulation. This no-code approach including a domain metamodel, a graphical concrete syntax, and a model-to-text transformation has been developed. The simulated sensors, actuators, fog nodes, cloud nodes and analytical characteristics are created as microservices and docker containers where elements are connected by using a publish–subscribe communication protocol. Two examples for smart building and agriculture IoT environments are presented to show how the simulation system works.

The study in [10] discussed the experiences of applying the ThingML to different domains. ThingML is an open-source tool which provides a family of code generators for heterogeneous platforms. ThingML consists of a modeling language and tools to support code generation. In [11], the authors proposed a code generation framework CAPSml based on the CAPS modeling framework. Through a graphical user interface, the CAPS framework supports the creation of IoT system architectures. CAPSml transforms the CAPS model into ThingML, a code generation framework that brings MDE to the late design and implementation stages. In this way, the CAPS users can generate models without the knowledge of ThingML.

The study in [12] proposed Orcc-IoT, an open-source dataflow environment with IoT features. Orcc-IoT facilitates the development of IoT by combining dataflow modeling language, heterogeneous code generator and the library of ready-made IoT actors. Orcc-IoT addressed the issues of the inherent heterogeneity of IoT systems with the presence of short-range and wide-area network links. Orcc-IoT will be published as open-source software under the original Orcc license (BSD).

With different levels of hardware abstraction, security and programming language, the study in [13] presented a web application development that reduces the startup time of a project and the learning curve of a new user. The sensors are configured through a simple GUI. The combined pattern techniques were used to generate the code for the firmware to integrate the sensor nodes in an IoT architecture. Based on Xtext and Eclipse Modeling Framework, a toolset consisting of a domain-specific language was proposed to create a model of a network of things and an extensible code generator to create the network artifacts from this model.

The authors in [14] proposed AutoIoT to create IoT applications based on a user-driven MDE approach. To model an IoT system, AutoIoT allows a developer to use a simple JSON file to specify internal model-to-model and model-to-text transformations. Then, AutoIoT generates a ready-to-use IoT application.

The above approaches [10,11,12,13,14,17,18,19,20], as well as IFTTT and Samsung SmartThings, support LCNC code generation in the network domain only. LCNC for the device domain are not addressed by these approaches.

The study in [16] proposed an API client generator called cpp-tiny-client, which is developed as a plugin for the OpenAPI Generator project. This approach tailors the generated code based on the specified IoT platform, which allows the developers to generate the correct code for API clients of the IoT devices. The cpp-tiny-client mechanism is similar to the DA mechanism in IoTtalk, where the DA code for a controller of ESP family is automatically generated to connect to the IoTtalk server. Automatic generation of the DA code will be described in Appendix A.

## 3. The DeviceTalk Architecture

DeviceTalk is an extension of our previous work, IoTtalk [2]. Following the MDE process approach [10,14,15,18,20,21], IoTtalk is an IDE environment similar to [17]. IoTtalk defines an abstract model, *d*, for the same type of IoT devices. The IoT device model d is represented as a set ***S_d_***, and an element *e_d_* of the set is called a device feature (DF). A DF is called an input DF (IDF) $e_{d,I}$ if it is a sensor or a control (such as a button). A DF is called an output DF (ODF) $e_{d,O}$ if it is an actuator (such as a fan). The set $S_{d,I}$ of all $e_{d,I}$ in the IoT device model d is called the “input device” of d, and the set $S_{d,O}$ of all $e_{d,O}$ is called the “output device”. Therefore, we have $S_{d}=S_{d,I}\cup S_{d,O}$. If d is a smartphone, then $S_{d,I}$ is a set of sensors for acceleration, gyro and orientation, and controls such as s keyboard. Similarly, $S_{d,O}$ is a set of actuators including a display screen, a speaker and so on. Let D be the set of the IoT devices in a distributed intelligent system. Let $D_{I}=\left\{S_{d,I}|\forall d\in D\right\}$ and $D_{O}=\left\{S_{d,O}|\forall d\in D\right\}$. Then, the network program for the system is a non-linear mapping from $D_{I}$ to $D_{O}$. The above abstract network model is created by the IoTtalk GUI illustrated in Figure 1. In this figure, the SoilSensor device is represented by an icon (Figure 1(1b)), where $S_{SoilSensor,I}$ = {Moisture-I, EC-I, pH-I} and $S_{SoilSensor,O}=\Phi$. The mapping is established by the join links in Figure 1, creating the network application following the LCNC approach described in Section 1.

**Figure 2:**
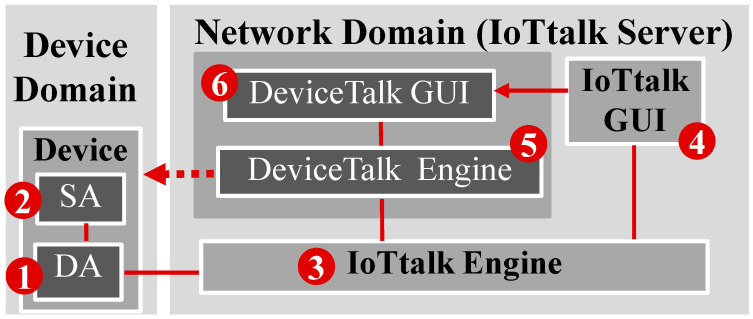
The DeviceTalk architecture for client–server-based intelligent distributed system.

IoTtalk also provides a simple mechanism to connect the abstract model to the real device. To do so, the real device must be equipped with the specific software model DA (Figure 2(1)) to communicate with the IoTtalk server. The IoT device needs another software module SA (Figure 2(1)) to implement the logic of the device (for example, the acceleration sensor algorithm). IoTtalk assumes that the application developer already implemented the SA and the DA programs in the IoT devices before they are accommodated in the distributed intelligent system, i.e., connected to the IoTtalk engine (Figure 2(3)). With the developer’s inputs, DeviceTalk will automatically generate the SA/DA codes as follows. We first create the device icon (for example, Figure 1(1b)) from the IoTtalk GUI (Figure 2(4)). Note that how the DFs are included in a device icon (e.g., Figure 1(1b,2b)) is not described in this paper and can be found in [2]. When the device icon is created, $S_{d}$ is defined and is stored in the IoTtalk engine. In the “Project” window (Figure 1), there is a “Save & Create SA Code” button. When this button is pressed, $S_{d}$ is sent from the IoTtalk engine to the DeviceTalk engine (Figure 2(5)), and the DeviceTalk GUI window (Figure 2(6)) pops up to allow the developer to create the SA/DA codes through an LCNC setup procedure using $S_{d}$. In the current implementation, DeviceTalk can generate the SA/DA codes tailored for Arduino and Raspberry Pi and the general codes for C++ and Python.

As an example, consider the SoilSensor device model written in Python. When the “Save & Create SA Code” button is pressed, the IoTtalk Engine instructs the DeviceTalk Engine (through the path (3) → (5) in Figure 2) to use ***S_d_*** to generate the template SA/DA code $C_{SoilSensor}^{*}$ for $S_{SoilSensor},$ which is listed below:

01. import time
02. import DA
03. import ‘to-be-filled_0’
04. ServerURL = ‘to-be-filled_1’
05. Reg_addr = ‘to-be-filled_2’
06. DA.profile[‘dm_name’] = ‘SoilSensor’
07. DA.profile[‘df_list’] = [‘Moisture-I’, ‘EC-I’, ‘pH-I’,]
08. DA.profile[‘d_name’] = ‘to-be-filled_3’
09. DA.register(ServerURL, Reg_addr)
10. while True:
11.   try:
12.     Moisture_data = to-be-filled_4
13.     DA.push (‘Moisture-I’, Moisture_data)
14.     EC_data = to-be-filled_5
15.     DA.push (‘EC-I’, EC_data)
16.     pH_data = to-be-filled_6
17.     DA.push (‘pH-I’, pH_data)
18.   except Exception as e:
19.     print(e)
20.     if str(e).find(‘mac_addr not found:’) != −1:
21.      print(‘Reg_addr is not found. Try to re-register.’)
22.      DA.register (ServerURL, Reg_addr)
23.     else:
24.      print(‘Connection fails.’)
25.      time.sleep(1)
26.   time.sleep(to-be-filled_7)

Lines 1 and 2 of $C_{SoilSensor}^{*}$ import the libraries to be used for the SA code. We will elaborate on the details of the DA library in Appendix A.

The DeviceTalk GUI (Figure 2(6)) enables the developer to complete the “to-be-filled” parts, including the target device name in “to-be-fill_3” (e.g., snsr1). Then, DeviceTalk translates $C_{SoilSensor}^{*}$ to the SA code $C_{snsr1}$. Finally, the developer uploads $C_{snsr1}$ and installs it into the real IoT device snsr1.

## 4. The DeviceTalk Procedures

**Figure 3:**
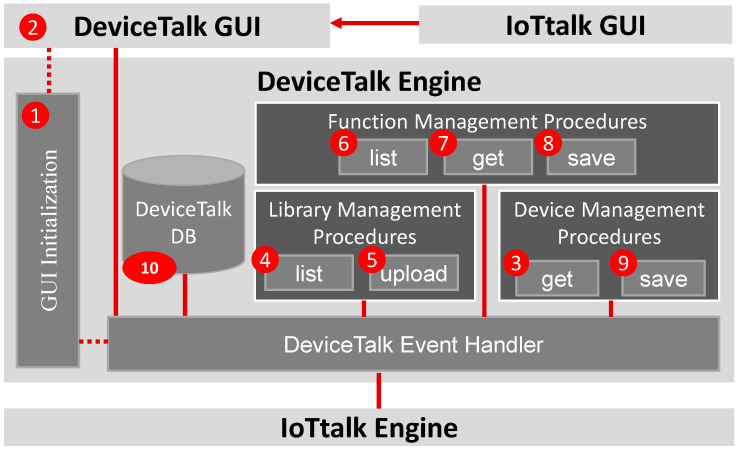
The procedures of the DeviceTalk Engine.

**Figure 4:**
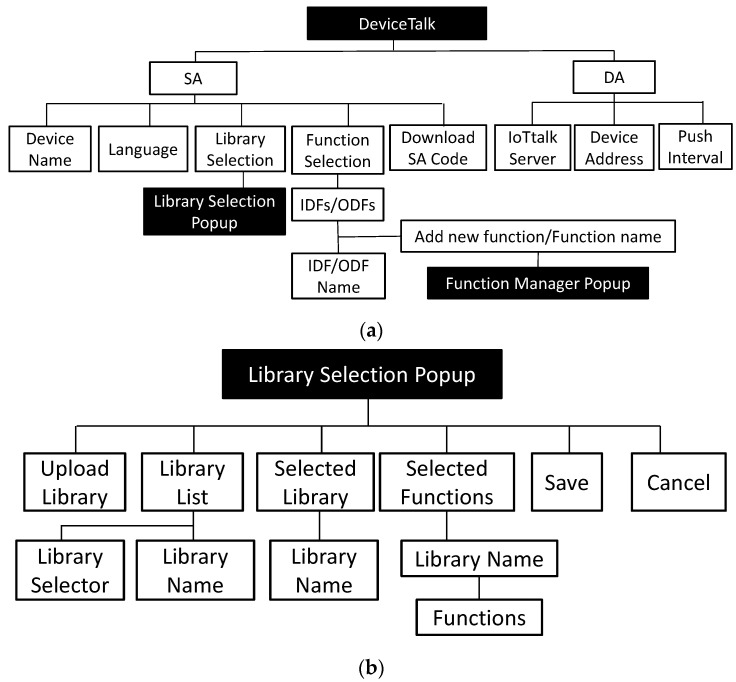

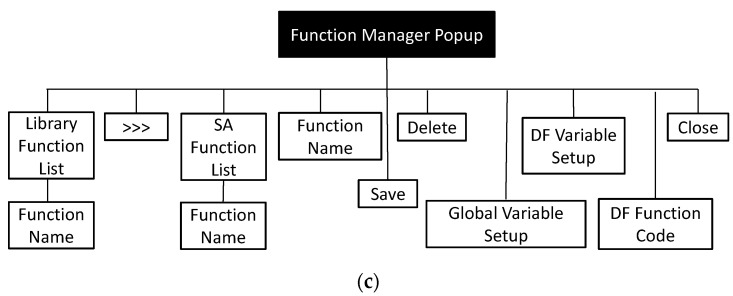
The structure of the DeviceTalk GUI. (**a**) The main GUI structure; (**b**) The structure of the Library Selection popup window; (**c**) The structure of the Function Manager popup window.

This section describes how the “to-be-filled” parts of $C_{SoilSensor}^{*}$ are complete through the procedures executed in the DeviceTalk Engine. Figure 3 provides the DeviceTalk Engine details of Figure 2(5), and Figure 4a shows the main structure of the DeviceTalk GUI (Figure 2(6)).

**Figure 5:**
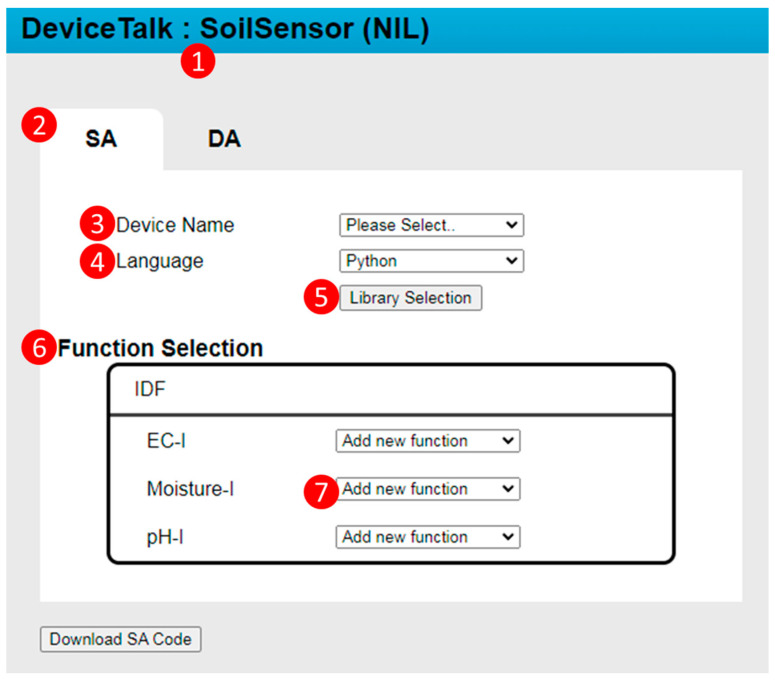
The DeviceTalk GUI.

Suppose that the developer wants to create the SA/DA code for a device called snsr1, which is derived from the device model SoilSensor. The developer selects SoilSensor from the “Model” list of IoTtalk GUI (Figure 1(5)). If the developer presses the “Save & Create SA Code” button after he/she has set up the device model (Figure 1(1b)), then the GUI (Figure 2(4)) instructs the IoTtalk Engine (Figure 2(3)) to provide the SoilSensor’s $S_{d}$ to the DeviceTalk Engine (Figure 2(5)). Then, Procedure “GUI Initialization” (Figure 3(1)) is executed to instruct the DeviceTalk GUI (Figure 3(2)) to show the webpage layout following the structure defined in Figure 4. The root of GUI has two branches—the SA and the DA tabs—and its layout is illustrated in Figure 5. The title bar (Figure 5(1)) specifies the device model name “SoilSensor”. Before the actual device name is given, the title bar indicates “NIL”.

In the SA tab (Figure 5(2)), when the developer selects “snsr1” in the “Device Name” pulldown list (Figure 5(3)), “NIL” is replaced by “snsr1” in the title bar, and Procedure “device-get” (Figure 3(3)) is executed to retrieve the metadata $M_{snsr1}$ of this device from the DeviceTalk DB (Figure 3(10)). The metadata $M_{snsr1}$ listed in m01–m20 is sent to DeviceTalk GUI and maintained by the “Vue.js” front-end framework.

m01. {

m02.  SA: {

m03.   dm_name: “SoilSensor”,

m04.   d_name: “snsr1”,

m05.   language: “Python”,

m06.   lib_selection: [<library>, …],

m07.   idfs: [

m08.    {

m09.     name: “Moisture-I”,

m10.     function: <SA function>

m11.    },

m12.    …

m13.   ],

m14.   odfs: [],

m15.   global_variable: “…”

m16.  },

m17.  DA: {

m18.   //to be elaborated in d01–d06 later

m19.  }

m20. }

Note that “snsr1” can be an existing device or a new device to be created. When the developer selects the language (e.g., Python in Figure 5(4)), the selected language is assigned to the “language” variable in Line m05, and DeviceTalk GUI will update the language information in $M_{snsr1}$.

**Figure 6:**
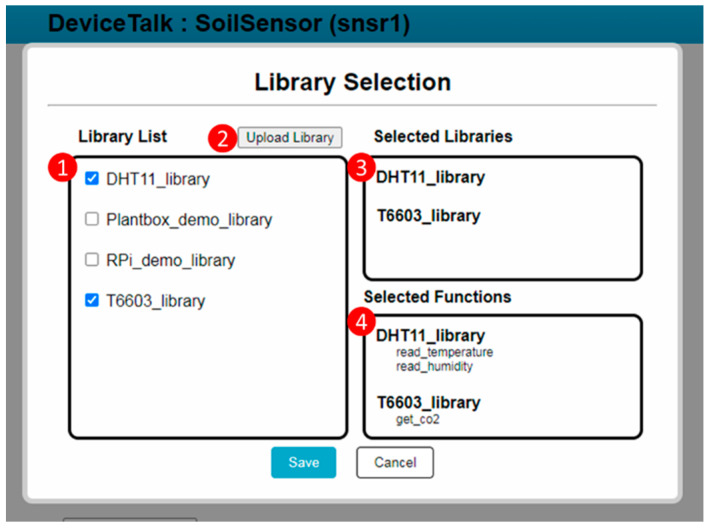
The library window.

The developer needs to create the functions for the DFs (i.e., Moisture_data, EC_data and pH_data in Lines 12, 14 and 16 of $C_{SoilSensor}^{*}$). He/she can write new functions or select the functions from existing libraries. The latter case is achieved by pressing the “Library Selection” button (Figure 5(5)). When this button is pressed, the “Library Selection” window (Figure 6) pops up. Figure 4b illustrates the GUI layout structure of the “Library Selection” window. Procedure “library-list” (Figure 3(4)) is executed to retrieve the library list of the DF to be shown in the “Library List” box (Figure 6(1)). Note that many off-the-shelf sensor/actuator products provide the driver codes that can be downloaded to drive the IoT hardware in the control boards such as Arduino and Raspberry Pi. DeviceTalk allows the developer to upload such driver code as a library (Figure 6(2)) by executing Procedure “library-upload” (Figure 3(5)). This procedure enables the developer to select a directory from his/her local computer. Then, all functions of the files under the directory are stored in the DeviceTalk DB (Figure 6(1)). When the potential libraries to be used by the DF are chosen, they are listed in the “Selected Libraries” box (Figure 6(3)), and Procedure “function-list” (Figure 3(6)) is executed to list all functions in these libraries in the “Selected Functions” box (Figure 6(4)) for readability. When the “Save” button is pressed, Line 6 of ***M_snsr_*_1_** is updated and the selected libraries are stored.

**Figure 7:**
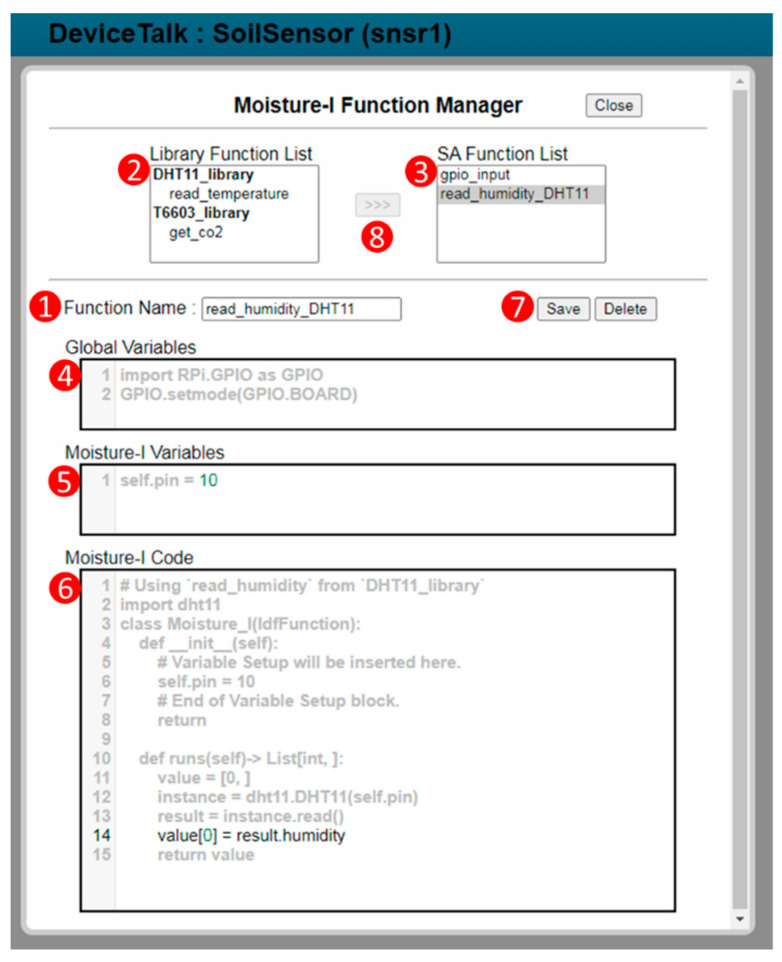
DF Function Manager.

After the libraries for snsr1 have been selected, the developer assigns the functions to the DFs through the “Function Selection” box (Figure 5(6)). When a function for Moisture-I is selected from its function list (Figure 5(7)), Moisture-I “Function Manager” window (Figure 7) pops up. The layout of this window is illustrated in Figure 4c. DeviceTalk provides a template for function code creation in the code area (Figure 7(6)), and the parts that should not be modified are marked gray. The details are given in Appendix B.

If the “Add new function” item is selected in Figure 5(7), then the developer fills the “Function Name” field (Figure 7(1)) to create a new function. On the other hand, if an existing function is selected, the name of the selected function is shown in Figure 7(1). The “Library Function List” (Figure 7(2)) is the same as the “Selected Functions” list in Figure 6(4). Through the function selector (Figure 7(8)), the developer can select the functions (e.g., “read_humidity_DHT11”) from this list and include them in the “SA Function List” for the DF (Figure 7(3)). To do so, Procedure “function-list” (Figure 3(6)) is executed to update the Moisture-I function list when the “read_humidity” SA function is included. When a function is selected in Figure 7(3), Procedure “function-get” (Figure 3(7)) is executed to retrieve the related information from DeviceTalk DB and show them in the variable windows (Figure 7(4,5)) and the code window (Figure 7(6)). Typically, the developer only modifies the global or the DF’s variables (Figure 7(4,5)) if needed. The existing function code (Figure 7(6)) is seldom modified, which is often reviewed by the developer to confirm that the correct function is selected. DeviceTalk provides a template for creating the program in the code area. The details for manipulating the function code are given in Appendix B. After the “Save” button (Figure 7(7)) is clicked, Procedure “function-save” (Figure 3(8)) is executed to update the function content (variables and function code) in DeviceTalk DB, and the function name will be stored in the metadata $M_{snsr1}$; for example, in Line m10, Moisture_data is assigned the “read_humidity” function.

**Figure 8:**
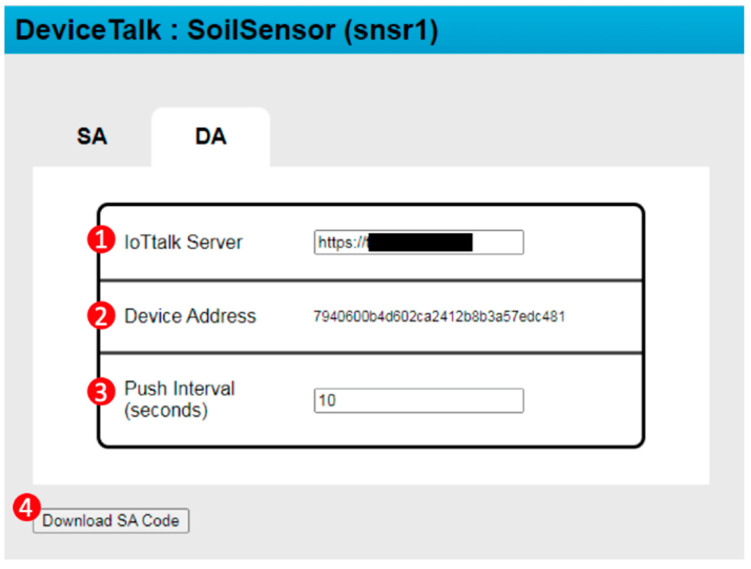
The DA window.

After all DFs have assigned their SA functions and the SA setup is completed, the developer can flip from the SA tab (Figure 5(2)) to the DA tab (Figure 8). In this tab, the “IoTtalk Server” field (Figure 8(1)) specifies the URL for the IoTtalk server to be connected by this device, “Device Address” field (Figure 8(2)) is automatically generated, and the “Push Interval” (in seconds; Figure 8(3)) specifies the data sampling frequency for snsr1. Procedure “device-get” (Figure 3(3)) stores the above DA information in Lines m17–m19 of $M_{snsr1}$, where the details are given in Lines d01–d05 below:

d01. DA: {

d02.  iottalk_server: “the IoTtalk server URL”,

d03.  device_address: “7940600b4d6…”,

d04.  push_interval: 10

d05. }

The above code is used to create the DA code described in Appendix A.

**Figure 9:**
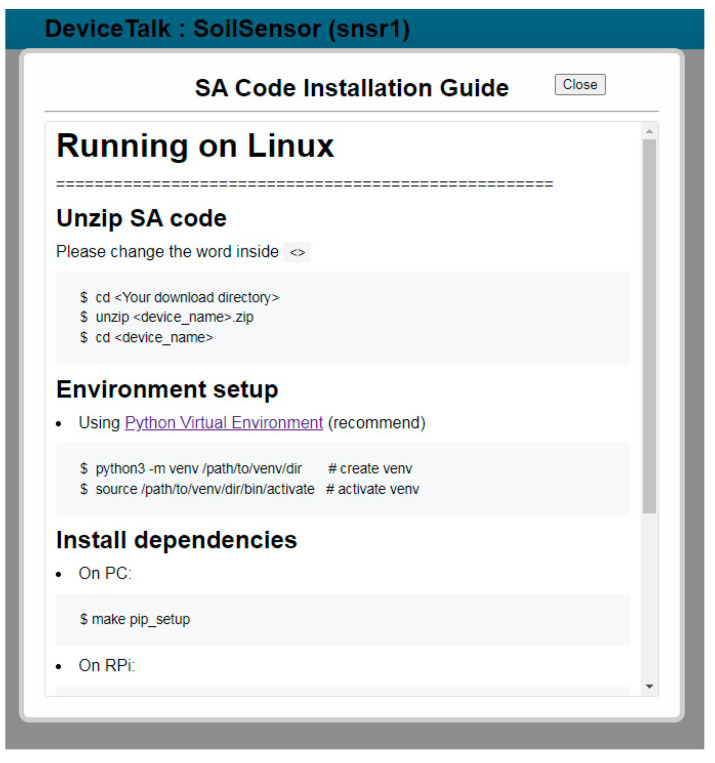
The SA Code Summary window.

After both SA and DA setups are completed, the developer clicks the “Download SA/DA Code” button (Figure 8(4)), and Procedure “device-save” (Figure 3(9)) is executed to retrieve the device information in DeviceTalk DB to fill the “to-be-filled” parts of $C_{SoilSensor}^{*}$. This procedure also uses the DA template $D_{SoilSensor}^{*}$ (see Appendix A) and the DA parts of $M_{snsr1}$ to produce the DA code $D_{snsr1}$. At the same time, a new library is created; this is the collection of the global variables (listed in Line m15) and all SA functions used by the DFs of the device. This new library is named “<device_name>_library” (e.g., “snsr1_library” in our example), and is stored in DeviceTalk DB for future usage. Finally, a zip file which is the collection of $C_{snsr1}$, DA file $D_{snsr1}$ and all the required libraries will be downloaded automatically, and DeviceTalk pops up the “SA Code Installation Guide” window (Figure 9) to show how to install $C_{snsr1}$ and $D_{snsr1}$ into the IoT device.

Note that for a sensor (e.g., humidity), there are a diverse set of hardware models, and their corresponding functionalities are different. The user must select the correct function for the sensor hardware he/she uses. The creation of these functionalities is achieved through the GUI operations in Figure 5(7), Figure 6 and Figure 7(6). In IoTtalk, for example, we use the humidity hardware model DHT 11 and the CO_2_ hardware model T6603 (see Figure 6(3)). Therefore, the function library for a hardware sensor must be named by its model number when the library is included in DeviceTalk. When a no-code user creates the SA for DHT11 humidity sensor, he/she will select DHT11_library using operation (8) in Figure 6.

## 5. Discussion and Conclusions

**Figure 10:**
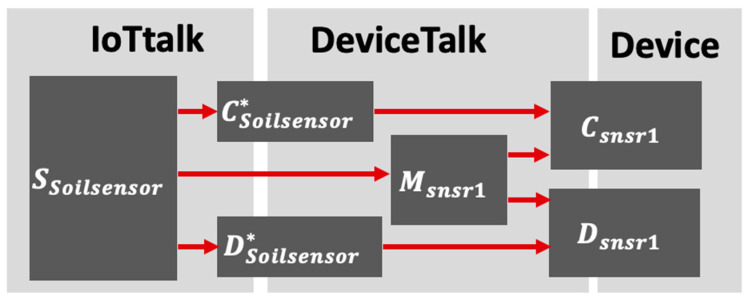
The SA/DA code generation for the snsr1 device from the SoilSensor device model.

The LCNC approaches for IoT application development are very advantageous in improving the speed of production; they have gained a lot of momentum in recent years and are even close to replacing traditional programming approaches. Most of the LCNC approaches target code development in the network domain. In this paper, we proposed DeviceTalk for code generation in the device domain. We showed how the specification of a real IoT device is generated from the IoTtalk GUI, and how DeviceTalk creates the SA and the DA codes for the device. We showed that the DA code can be automatically generated by DeviceTalk without manual involvement, and can be set up through a no-code approach. In summary, the code generation process is illustrated in Figure 10. The SA/DA code for the snsr1 device is generated from the SoilSensor device model as follows: $S_{soilsensor}$ is abstracted from the device model icon specified by the developer through the IoTtalk GUI. The IoTtalk Engine automatically generates the $C_{Soilsensor}^{*}$ and the $D_{Soilsensor}^{*}$ codes from $S_{soilsensor}$. The $M_{snsr1}$ code is generated from $S_{soilsensor}$ with the developer’s setup through the DeviceTalk GUI. Then, the DeviceTalk Engine automatically generates the $C_{snsr1}$ code from $M_{snsr1}$ and $C_{Soilsensor}^{*}$. Similarly, the $D_{snsr1}$ code is automatically generated from $M_{snsr1}$ and $D_{Soilsensor}^{*}$.

The SA code is generated by the no-code approach if the code for driving the sensor/actuator hardware already exists in DeviceTalk. Note that many off-the-shelf sensor/actuator products provide the software that can be downloaded to drive the IoT hardware in the control boards such as Arduino and Raspberry Pi. Therefore, the developer can download the sensor/actuator software modules and save them in the DeviceTalk library. Through the off-the-shelf driver code, DeviceTalk supports a LCNC approach to assist the developer to translate the driver code to the SA function code (for the sensor/actuator) to be executed in the IoT device (e.g., the Arduino control board). After the SA function code has been created, it can be saved in DeviceTalk, and is used to automatically generate the SA code for the target IoT device. If the developer uses the same sensor/actuator in another IoT device later, he/she can select the existing SA function code without writing any code through the DeviceTalk no-code mechanism. Based on the LCNC paradigm, DeviceTalk speeds up the code development in the device domain for an intelligent distributed system.

We have tested our approach to ensure that the automatically created SA/DA codes are correct. The LCNC approach of IoTtalk network applications supports embedded systems such as MediaTek LinkIt Smart 7688 duo, ROHM IoT kit and ESP8266 (NodeMCU) with the same DA software [22]. We also support Raspberry Pi. Consider the intelligent hydroponic plant box as an example [23]. Let $C_{plant}^{DT}$ be the SA code generated by DeviceTalk and $C_{plant}^{m}$ be the code manually generated by an experienced programmer. The size of $C_{plant}^{DT}$ is 193 lines and the size of $C_{plant}^{m}$ is 133. DeviceTalk generates 60 more lines because it generates the SA using Python classes, where declaration and invocation of a library class incurs extra cost. The advantage is that the library codes of $C_{plant}^{DT}$ can be reused and are easy to debug in the modular way. Consider the color light of the plant box. The Color-O ODF code segment for $C_{plant}^{m}$ is (13 lines).

01. red_pin = 19
02. green_pin = 21
03. blue_pin = 23
04. GPIO.setup(red_pin, GPIO.OUT)
05. GPIO.setup(green_pin, GPIO.OUT)
06. GPIO.setup(blue_pin, GPIO.OUT)
07. def color(value):
08.   red_value = 1 if value[0] > 0 else 0
09.   green_value = 1 if value[1] > 0 else 0
10.   blue_value = 1 if value[2] > 0 else 0
11.   GPIO.output(red_pin, red_value)
12.   GPIO.output(green_pin, green_value)
13.   GPIO.output(blue_pin, blue_value)

The Color-O ODF code segment for $C_{plant}^{DT}$ is (19 lines).

01. class Color_O(OdfFunction):
02.   def __init__(self):
03.     # Variable Setup block
04.     self.red_pin = 19
05.     self.green_pin = 21
06.     self.blue_pin = 23
07.     # End of Variable Setup block.
08.     GPIO.setup(self.red_pin, GPIO.OUT)
09.     GPIO.setup(self.green_pin, GPIO.OUT)
10.     GPIO.setup(self.blue_pin, GPIO.OUT)
11.     return
12.   def runs(self, value):
13.     red_value = 1 if value[0] > 0 else 0
14.     green_value = 1 if value[1] > 0 else 0
15.     blue_value = 1 if value[2] > 0 else 0
16.     GPIO.output(self.red_pin, red_value)
17.     GPIO.output(self.green_pin, green_value)
18.     GPIO.output(self.blue_pin, blue_value)
19.     return

To compare the performance of $C_{plant}^{DT}$ and $C_{plant}^{m}$; we execute each of them for 50 times. For $C_{plant}^{m}$, the average processing time is 1.436 s, the maximum time is 1.505 s and the minimum time is 1.405 s. For $C_{plant}^{DT}$, the average processing time is 1.432 s, the maximum time is 1.482 s and the minimum time is 1.405 s. For the processing time performance, both $C_{plant}^{DT}$ and $C_{plant}^{m}$ are about the same. The average memory usage for $C_{plant}^{m}$ is 50.883 KB, the maximum usage is 50.9 KB and the minimum usage is 50.88 KB. The memory usage for $C_{plant}^{m}$ is fixed, which is 50.888 KB. Through the analysis of ANOVA (Analysis of variance), the *p*-value for the processing time is 0.421 (>0.05) and the *p*-value for the memory usage is 0.994 (>0.05).

The IoTtalk/DeviceTalk will become open source tools under the support of the Ministry of Education, Taiwan.

DeviceTalk does have its limitations. When a hardware sensor is first accommodated in DeviceTalk, the application developer does need to have some knowledge of the sensor to create the SA function. For any off-the-shelf sensor product, the manufacturer will provide the driver code and the example program to use the sensor. As described in Appendix B, after the SA function has been built following the example program provided by the manufacturer, the subsequent application developers who use this sensor product can create the SA/DA code by selecting the SA function with the no-code approach. Therefore, it would be better that the first developer who creates the SA function has the experience to find the driver codes for sensor products (through the low-code approach).

In the future, we will perform usability studies among different groups of no-code or low-code users. We will also use different groups of IoT devices and measure the complexity of application development with DeviceTalk. We have also compared the automated generated SA/DA codes with the codes manually generated by the IoTtalk experts. The amount of codes generated are the same. In the future, we will continue to observe the efficiency of code generation.

## Figures and Tables

**Figure A1:**
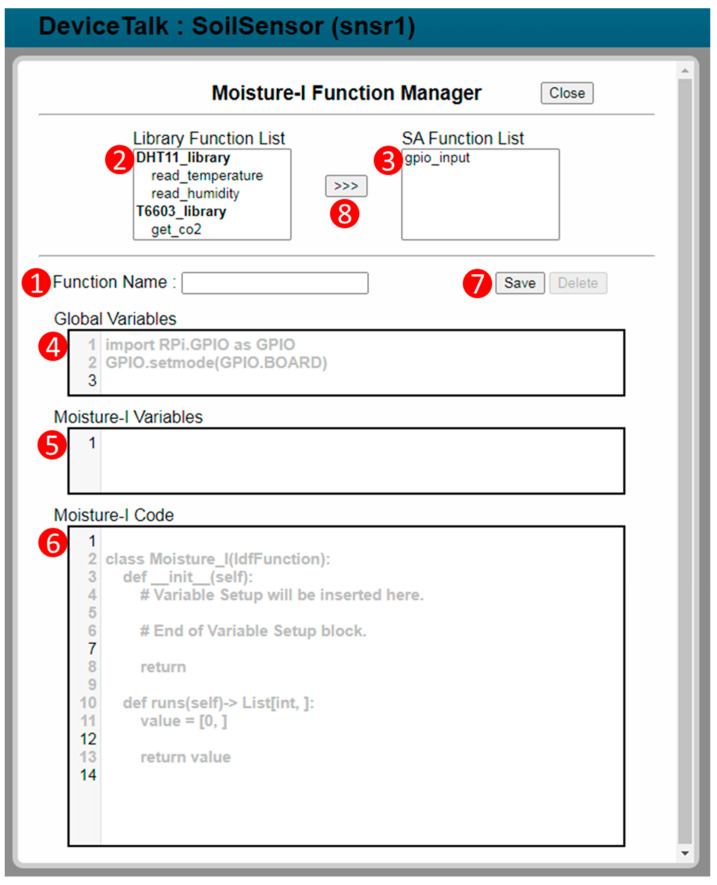
SA Function Template.

**Figure A2:**
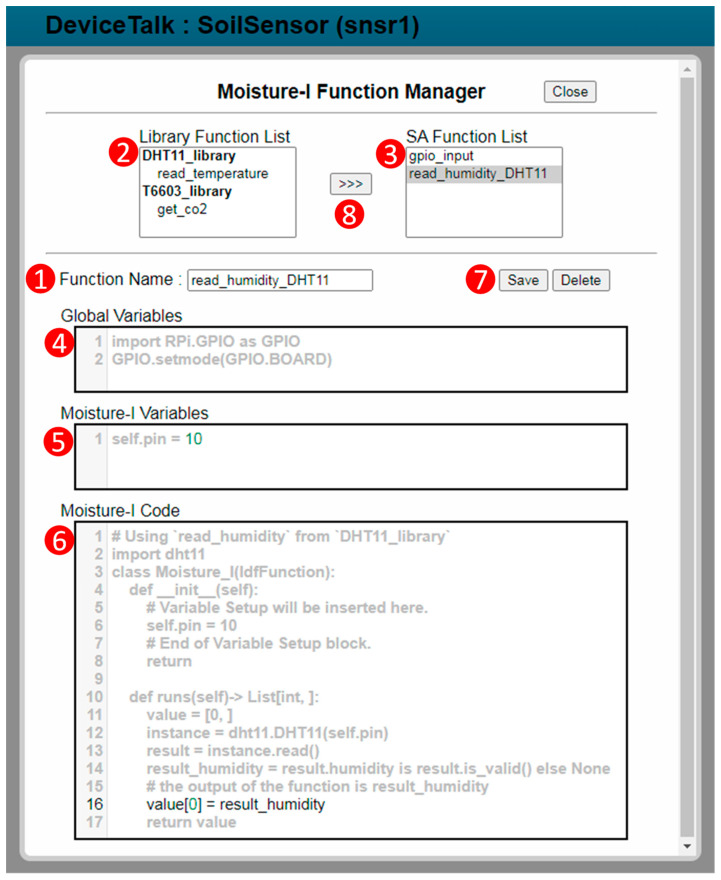
Example of the completed SA Function code.

## Data Availability

Not applicable.

## Appendix A. The DA Code in Python

This section elaborates on the DA library implementation. In our design, the DA library is generic and can be reused by all IoT devices. Use snsr1 as an example. Parts of DA library python code $D_{Soilsensor}^{*}$ are shown below:

01. import requests
02. profile = {
03.   ‘dm_name’: ‘SoilSensor’,
04.   ‘d_name’: None,
05.   ‘df_list’: [],
06. }

Line 1 imports the *requests* library, a popular HTTP protocol library in Python. Lines 2–6 declare the device’s metadata *profile*, which can be filled from the SA of the target IoT device.

07. endpoint = None
08. def register(ServerURL, reg_addr):
09.   global endpoint
10.   endpoint = ServerURL + ‘/’ + reg_addr
11.   r = requests.post(
12.    endpoint,
13.    json = {‘profile’: profile}
14.   )
15.   if r.status_code != 200:
16.    raise Error(r.text)
17.   return r.json().get(‘d_name’)

Line 8 defines the *register*() function, which is used to register the target device (snsr1 in our example) to the IoTtalk Engine. The IoTtalk Engine is designed in RESTful API style that can perform different operations using different HTTP methods with the same URL. Additionally, the IoTtalk Engine uses the device address in the endpoint (line 10) to identify the device. Lines 11–14 use the HTTP POST method to perform the registration procedure in the IoTtalk Engine with the metadata *profile*. After the device has been successfully registered, line 17 returns the registered device name.

18. def pull(feature_name):
19.   r = requests.get(
20.    endpoint + ‘/’ + feature_name
21.   )
22.   if r.status_code ! = 200:
23.    raise Error(r.text)
24.   return r.json().get(‘data’)

Line 18 defines the *pull*() function, which is used to obtain the data of a specified ODF from the IoTtalk Engine. Lines 19–21 invoke an HTTP GET request with the *feature_name* to query the corresponding data from the IoTtalk engine. Lines 22 and 23 raise an error if it fails, otherwise Line 24 returns the data.

25. def push(feature_name, data):
26.   r = requests.put(
27.    endpoint + ‘/’ + feature_name,
28.    json = {‘data’: data}
29.   )
30.   if r.status_code != 200:
31.    raise Error(r.text)
32.   return True

Lines 25–32 show the code for the *push*() function, which allows the IoT device to send data of a specific IDF to the IoTtalk Engine. In RESTful API style, the HTTP PUT method is used to update the information. Lines 26–29 pack the data in JSON format and send the IDF data to the IoTtalk Engine. Line 32 returns True if the push operation is executed successfully. In the DA tab, DeviceTalk uses $D_{Soilsensor}^{*}$ and the developer inputs to produce the DA code $D_{snsr1}$.

## Appendix B. The SA Function Code Creation

This appendix describes how the SA function code is developed in the “DF Function Manager” (Figure 7(5,6)). If the developer selected “Add new function” in Figure 5(7), DeviceTalk generates the template of SA function code shown in Figure A1(6). The template lines are marked gray and should not be modified to make sure that the SA function’s return type is the same as the DF parameter’s type.

Lines 3–8 of Figure A1(6) define the constructor of the SA function. All sensor-based variables declared in Figure A1(5) (to be filled by the developer) will be inserted in Lines 4–6 automatically. All global variables are declared in Figure A1(4), which are shared by all DFs in this device. The developer should only modify the DF’s variables in Figure A1(4,5) when the function is reused later. Lines 10–13 of Figure A1(6) define the *runs* function, which is the main part of this SA function.

The areas with the black line numbers in Figure A1(6) are filled by the developer. For example, Line 1 (or more) is filled if *runs* needs to import other library functions. Line 7 (or more) is filled if some instructions are executed in the constructor. Line 12 (or more) must be filled to implement the logic of the *runs* function. In particular, the variable *value* defined in Line 11 must be assigned in the body of *runs.* Lines 14 onwards are filled if more functions are included.

For any off-the-shelf sensor product, the manufacturer will provide the driver code and the example program to use the sensor. Use Moisture-I as an example. Suppose that the snsr1 device uses the humidity sensor product DHT-11, and we give the SA Function name “read_humidity_DHT11” (Figure A2(1)). We first download the driver and the example codes of the sensor in the DHT11_library. When we select the “read_humidity” function from Figure A2(2) and press the function selector (Figure A2(8)), the “read_humidity” function is moved to the “SA Function List” (Figure A2(3)) and automatically appends the library name (e.g., read_humidity_DHT11) to determine which library is used. Since the device is a Raspberry Pi, the related global variables are automatically filled in Figure A2(4). The “read_humidity” function in the DHT-11 library takes one argument, “*self.pin*”, and DeviceTalk automatically fill “*self.pin =*” in Figure A2(5). If the DHT11 sensor is connected to pin 10 of Raspberry Pi, then the developer set up the variable “*self.pin = 10*”.

In the Moisture-I code area, the areas with the black line numbers in Figure A1(6) can be further filled automatically when “read_humidity” is selected. For example, Lines 1 and 2 import the library DH11_library. When the developer assigns a value to “*self.pin*” in Figure A2(5), DeviceTalk automatically fills the value in Line 6 of Figure A2(6). Lines 12–14 are directly copied from the example program of the DH11 sensor device. Line 15 provides the hint in the comment “the output of the function is result_humidity”. Then, in Line 16, the developer assigns the value result_humidity to value [0].

After the developer has created “read_humidity_DHT11”, this SA function is saved in DeviceTalk DB as part of the snsr1 library. DeviceTalk will also create a pointer in DHT11_library to link “read_humidity” to the SA function “read_humidity_DHT11” in the snsr1 library. When the next developer attempts to use the DHT11 sensor, he/she may include DHT11_library. When “read_humidity” is selected, the incomplete code in Figure A2(6) is not shown. Instead, the complete “read_humidity_DHT11” is shown in the code area of the “DF Function Manager”. Therefore, the next developer can reuse the code created for snsr1 directly without any modification.
